# Entomopathogenic nematodes in agricultural areas in Brazil

**DOI:** 10.1038/srep45254

**Published:** 2017-04-06

**Authors:** Andressa Lima de Brida, Juliana Magrinelli Osório Rosa, Cláudio Marcelo Gonçalves de Oliveira, Bárbara Monteiro de Castro e Castro, José Eduardo Serrão, José Cola Zanuncio, Luis Garrigós Leite, Silvia Renata Siciliano Wilcken

**Affiliations:** 1 Departamento de Proteção Vegetal, Universidade Estadual Paulista (UNESP/FCA), Rua José Barbosa de Barros, 1780, CEP 18610-307, Botucatu, São Paulo, Brasil; 2 Laboratório de Nematologia (CEIB), Instituto Biológico, Alameda dos Vidoeiros, 1097, CEP 13101-680, Campinas, São Paulo, Brasil; 3 Departamento de Fitotecnia, Universidade Federal de Viçosa, 36570-900, Viçosa, Minas Gerais, Brasil; 4 Departamento de Biologia Geral, Universidade Federal de Viçosa, 36570-900, Viçosa, Minas Gerais, Brasil; 5 Departamento de Entomologia/BIOAGRO, Universidade Federal de Viçosa, 36570-900, Viçosa, Minas Gerais, Brasil; 6 Laboratório de Controle Biológico (CEIB), Instituto Biológico, Alameda dos Vidoeiros, 1097, CEP 13101-680 Campinas, São Paulo, Brasil

## Abstract

Entomopathogenic nematodes (EPNs) (Steinernematidae and Heterorhabditidae) can control pests due to the mutualistic association with bacteria that kill the host by septicemia and make the environment favorable for EPNs development and reproduction. The diversity of EPNs in Brazilian soils requires further study. The identification of EPNs, adapted to environmental and climatic conditions of cultivated areas is important for sustainable pest suppression in integrated management programs in agricultural areas of Brazil. The objective was to identify EPNs isolated from agricultural soils with annual, fruit and forest crops in Brazil. Soil samples were collected and stored in 250 ml glass vials. The nematodes were isolated from these samples with live bait traps ([*Galleria mellonella* L. (Lepidoptera: Pyralidae) larvae]. Infective juveniles were collected with White traps and identified by DNA barcoding procedures by sequencing the D2/D3 expansion of the 28S rDNA region by PCR. EPNs identified in agricultural areas in Brazil were *Heterorhabditis amazonensis, Metarhabditis rainai, Oscheios tipulae* and *Steinernema rarum*. These species should be considered pest biocontrol agents in Brazilian agricultural areas.

Entomopathogenic nematodes (EPNs) Steinernematidae and Heterorhabditidae can control pests due to mutualistic association with bacteria of the genus *Xenorhabdus* (Thomas & Poinar) and *Photorhabdus* (Boemare, Louis & Kuhl), respectively^1^,^2^. These nematodes penetrate the host through natural openings or through the cuticle transporting bacteria into the hemocele^3^,^4^ where they reproduce and kill the host from septicemia within 24 to 48 hours^5^,^6^, making the environment favorable for nematode development and reproduction^7^. Infective juveniles seek another host in the soil when the insect host resources run out^8^. Interest in these biological control agents is increasing^9^ due to the reduced efficiency of conventional chemical and cultural methods for insect soil management and the broad spectrum of EPN hosts^10^.

EPNs are globally distributed, with different species and groups according to geographic regions^11^,^12^. Information on EPNs and their symbiotic bacteria is scarce in many countries, including in Brazil. *Heterorhabditis amazonensis* Andaló and *Steinernema brasiliensis* Nguyen were reported as native species in Brazil^13^,^14^ and *Heterorhabditids indica* and *H. baujardi* were reported in Rondônia state, Brazil^15^. Isolates of *H. amazonensis, H. baujardi, H. indica* and *H. mexicana* were found in Minas Gerais state, Brazil^16^,^17^.

Nematode species can be identified by molecular characterization based on sequencing of rDNA subunit (28S)^18^,^19^,^20^, because low morphological variation and similar characteristics within this group hamper identification^15^. Infectivity, environmental tolerance and suitability for commercial formulations vary between EPN isolates and species^21^,^22^ which can be used to control pests of various orders, such as Coleoptera^23^,^24^,^25^, Hemiptera^26^,^27^,^28^,^29^,^30^ and Lepidoptera^31^,^32^,^33^.

EPN identification, adapted to environmental and climatic conditions of cultivated areas is important for sustainable pest suppression in integrated management programs in agricultural areas of Brazil. The objective was to identify EPNs from agricultural soils with annual, fruit and perennial crops in Brazil.

## Methods

### Nematode collection

Soil samples were collected in agricultural areas in Barretos, Botucatu, Garça, São Manuel (São Paulo State), and Palotina (Paraná State), Brazil from 2010 to 2013. Thirty-seven samples were collected in areas with annual crops, [*Glycine max* (L.) Merrill, *Zea mays* (L.), *Avena sativa* (L.), *Saccharum officinarum* (L.) and irrigated *Oryza sativa* (L.)], 20 in areas with forest plantations [*Anadenanthera falcata* (Benth.) Speg.), *Peltophorum dubium* (Spreng.) Taub., *Khaya ivorensis, Swietenia macrophylla, Hevea brasiliensis* (L.), *Eucalyptus* spp., *Cedrela odorata* (L.), *Acrocarpus fraxinifolius, Azadirachta indica* (A. Juss.), *Cordia ecalyculata* (Vell), *Calophyllum brasiliense* (Cambess.) and *Poecilanthe parviflora* (Benth)], and 97 in areas with fruit [*Litchi chinensis* (Sonn.), *Macadamia integrifolia* (Maiden & Betche), *Citrus reticulata* (L.), *Prunus persica* (L.), *Prunus* sp., *Psidium guajava* (L.), *Mangifera indica* (L.), *Citrus sinensis* (L.), *Citrus* sp., *Rubus idaeus* (L.), *Musa* spp. and *Coffea arabica* (L.)]. In addition, four samples were collected in native forest, one in a pasture and 42 in plowed soil areas, totaling 201 samples.

A zero to 25 cm deep soil sample was taken per sampling point and placed in 2L labeled plastic bags, stored in a Styrofoam box and transferred to the laboratory. The geographical coordinates of each sample were obtained with Garmin GPS device Etrex Vista H 2.8. Nematodes were isolated in the laboratory using fifth instar *Galleria mellonella* Linnaeus (Lepidoptera: Pyralidae) larvae. Briefly, each soil sample was packed into a 250 mL a glass vial with five *G. mellonella* larvae. These vials were covered and stored without light at 25 ± 2 °C. After three to seven days, dead *G. mellonella* showing nematode infection symptoms were removed, rinsed in distilled water and transferred to White traps^34^. The infective juveniles (IJs) were again inoculated in *G. mellonella* larvae for multiplication. Five *G. mellonella* larvae were used in a Petri dish (9 cm diameter) containing two moistened paper filters with 1.5 mL of solution with 100 JIs/larvae, for each nematode species. The samples were covered with PVC plastic and stored at 25 ± 2 °C and RH > 80%^35^. After three days, dead larvae were transferred to a White trap^34^ at 25 ± 2 °C for seven and 15 days. The IJs that left the *G. mellonella* carcasses were collected with distilled water every two days and stored at 18 °C. The nematode samples were named FCA 01 to 14 and stored in the Entomopathogenic Nematode Bank of the Nematology Laboratory at FCA/UNESP. Vials contained 1 M NaCl solution and were frozen at −80 °C until DNA extraction and EPN identification.

### PCR

Genomic DNA was obtained for each population (FCA 01 to 14) from three individuals of each EPN, extracted using Lysis Buffer Holterman [(HLB) (800 μg proteinase K/ml, β-mercaptoethanol 1% (v/v), 0.2 M NaCl and 0.2 M Tris HCl pH 8)]^36^. A total of 25 μL of HLB was diluted in 25 μL of ultrapure water totaling 50 μL in a 0.2 mL Eppendorff tube. A drop of this solution (5 μL) was placed on a glass slide, where the nematodes were individually cut into three parts and placed in the same 0.2 mL tube. The 45 μL of remaining solution was used to wash the slide and added to the tube with the sectioned nematode. Samples were submitted to PCR at 65 °C for 2 h, 99 °C for five minutes and stored at −20 °C^37^. The universal primers D2A (5′-CAAGTACCGTGAGGGAAAGTTG-3′) and D3B (5′TCGGAAGGAACCAGCTACT A-3′) were used to amplify the D2/D3 expansion segment of 28S rDNA by PCR^38^. A total of 12.5 μL of Gotaq Hot Start (Promega, São Paulo State, Brazil), with the reagents necessary for reaction: 5 U/μL Taq, 100 mM of each NTP and 25 mM MgCl_2_, 9.5 μL of nuclease free water (Promega), and 1 μL of each primer [10 mM] and 1 μL of cDNA from each representative population of target and non-target species, totaling 25 μL per reaction was submitted to pCR at 94 °C for seven minutes; followed by 35 cycles at 94 °C for 60 seconds, 55 °C for 60 seconds, 72 °C for 60 seconds; and 72 °C for 10 minutes^39^. Five μL of PCR product was used for electrophoresis in TAE buffer^40^ on 1% agarose gel, stained with ethidium bromide (0.02 mg/mL), visualized and photographed under UV light. The result of the amplification was compared to the molecular weight marker VIII.

The amplified fragments of D2/D3 expansion 28S rDNA were sequenced with the Big Dye Terminator kit (Applied Biosystems)^41^. A reagent mix containing 2 μL Big Dye, 3.2 mmol sense primers, 3.0 μL of amplified product containing 400 ng DNA and 2.0 mL of water was prepared for the product end of the PCR reaction. The reaction for sequencing was carried out according to manufacturer’s instructions (Applied Biosystems) with further purification of the amplified product by precipitation with isopropanol. Samples were denatured at 95 °C for three minutes and electrophoresis performed in an ABI Prism 377 DNA Sequencer unit (Applied Biosystems).

The sequences were aligned and compared to nucleotide polymorphism identification with the aid of BioEdit Aligment Sequence Editor Program. The EPN population sequences were compared with other nematode species in the database (GenBank, http://www.ncbi.nlm.nih.gov) for identification based on genetic similarity.

For phylogenetic analysis, the multiple alignments between the sequences of the region D2/D3 of the different isolates were edited manually with BioEdit Sequence Aligment program when phylogenetically uninformative columns were excluded from the analyses. Phylogenetic analyses were inferred using the Maximum Likelihood method based on the Kimura 2-parameter model^42^, considered the best fitting model for sequence evolution determined using the BIC scores (Bayesian Information Criterion) implemented in 6 MEGA program^42^. Initial trees for the heuristic search were obtained automatically by applying Neighbor-Join and BioNJ algorithms to a matrix of pairwise distances estimated using the Maximum Composite Likelihood (MCL) approach, and then selecting the topology with superior log likelihood value. A discrete Gamma distribution was used to model evolutionary rate differences among sites (4 categories (+*G*, parameter = 2.3432)). The model variation rate allowed for some sites to be evolutionarily invariable ([+*I*], 19.1558% sites). The tree was drawn to scale, with branch lengths measured in the number of substitutions per site. The analysis involved 48 nucleotide sequences, including 16 obtained in the present study (FCA01 to FCA16) and 32 from the GenBank. All positions containing gaps and missing data were eliminated. A total of 292 positions was obtained in the final dataset. Trees were sampled at intervals of 1000 generations and *Caenorhabditis elegans* (Brenner) was selected as outgroup. Evolutionary analyses were conducted in 6 MEGA program^42^.

## Results

**Figure 1:**
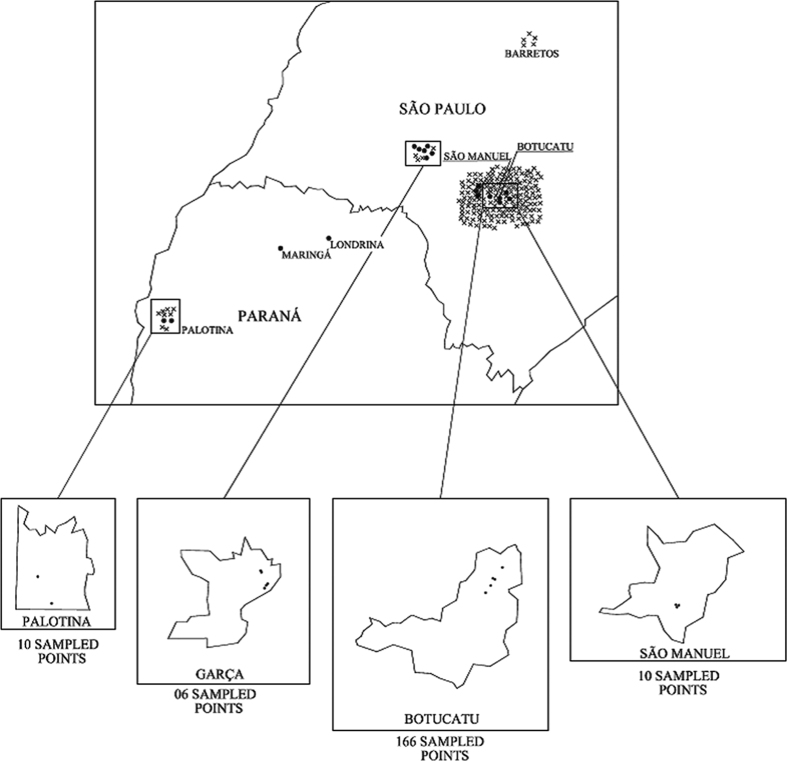
Geographical distribution of the sampling sites (x) in São Paulo and Paraná States, Brazil. EPNs positive samples (.) (ESC.: 1: 10000). AutoCAD SP2 (2015) [vJ.210.0.0]. http://www.Autodesk.com.br/products/autocad/overview.

The nematodes obtained from White traps were inoculated in new *G. mellonela* larvae, which demonstrates parasitism by the isolated entomopathogen. EPNs were found in 16 soil samples, corresponding to 8% of 201 samples. Seven (35%) of 20 samples from forest plantation areas had nematodes (FCA 04, FCA 05, FCA 06, FCA 07, FCA 08, FCA 10 and FCA 15). In annual crops, three (8.1%) out of 37 samples had nematodes: isolated FCA 11, detected in sandy soil in irrigated rice in Botucatu, São Paulo State, Brazil and isolates FCA 16 and FCA 03 in soybean crops in clay soils in Palotina, Paraná State, Brazil. Among 97 samples taken from orchards, six (6.2%) were positive for EPNs, FCA 12 isolates grown in sandy soils with wild raspberry, FCA 13 in citrus soil and FCA 14 in mango soil in São Manuel, São Paulo, Brazil. FCA 01 and FCA 02 isolates were also found in clay soil with citrus in Botucatu, São Paulo State, Brazil (Fig. 1). EPN samples were not found in plowed soil, native forest and pasture areas.

**Figure 2:**
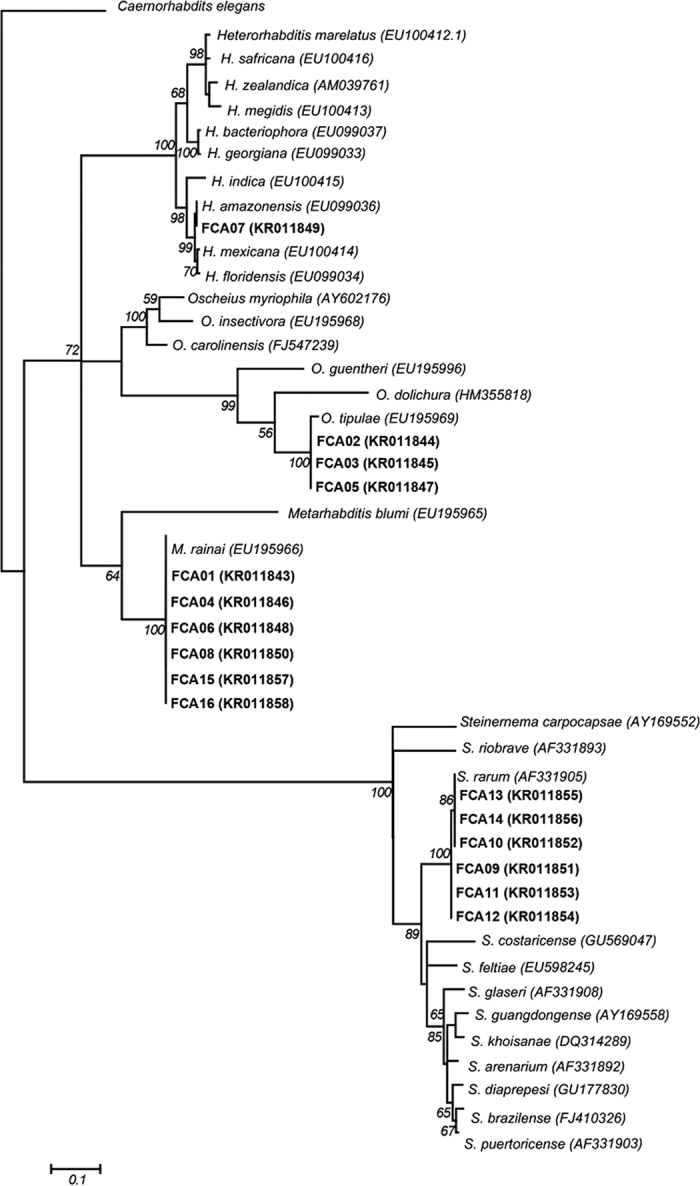
Phylogenetic tree showing the relationship between the entomopathogenic nematodes isolated (in bold) and their similarity with those from the GenBank based on expansion D2/D3 sequences 28S rDNA region. *Caenorhabditis elegans* was used as outgroup.

Amplification of D2/D3 expansion 28S rDNA gene of EPN isolates produced 590 bp fragments, whose sequences were deposited in the GenBank under the accession codes KRO11843 and KRO11858 (Fig. 2). The technique of DNA barcode sequences showed that the expansion D2/D3 28S rDNA gene of FCA 07 were identical to *H. amazonensis* (EU099036). The sequences of isolates FCA 01, FCA 04, FCA 06, FCA 08, FCA 15 and FCA 16 were identical to the *Metarhabditis rainai* (EU195966). The sequences of FCA 02, FCA 03 and FCA 05 isolates were identical to *Oscheius tipulae* (Lam & Webster, 1971) (EU195969). FCA 09, FCA 10, FCA 11, FCA 12, FCA 13 and FCA 14 isolates were observed (99–100%) with *Steinernema rarum* (AF331905). However, these isolates formed two groups that had three polymorphisms between them. One group with FCA 09, FCA 11 and FCA 12 isolates were similar to each other, but different in 3 bp from the group comprising FCA 10, FCA 13 and FCA 14 isolates. The phylogenetic tree (Fig. 2) obtained from the 48 aligned sequences of the D2/D3 expansion 28S rDNA genes in EPNs showed four distinct groups, *H. amazonensis* (FCA 07)*, S. rarum* (FCA 09, FCA 10, FCA 11, FCA 12, FCA 13 and FCA 14), *M. rainai* (FCA 01, FCA 04, FCA 06, FCA 08, FCA 15 and FCA 16) and *O. tipulae* (FCA 02, FCA 03 and FCA 05).

*Heterorhabditis amazonensis* was found in clay soil with *P. parviflora* in Garça, São Paulo State, Brazil; *S. rarum* in clay soil with *Eucalyptus* sp. and *C. reticulata* in Botucatu, São Paulo State, Brazil and in sandy soil with *R. idaeus* (FCA 12) and *C. reticulata* (FCA 13) and *M. indica* (FCA 14) in São Manuel, São Paulo State, Brazil.

*Metarhabditis rainai* was detected in clay soils cultivated with *A. fraxinifolius, C. odorata, C. brasiliense* and *C. ecalyculata*, in Garça, São Paulo State, Brazil and in those with *C. reticulata*, in Botucatu, São Paulo State, Brazil. *Oscheius tipulae* was found in soils with *A. indica* and *C. reticulata* in Botucatu, São Paulo State, Brazil and in clay soils cultivated with *G. max*, in Palotina, Paraná State. Brazil (Table 1).

## Discussion

Nematology surveys with *G. mellonella* baiting technique are useful to detect Steinernematidae and Heterorhabditidae species as well as other rhabditids. The molecular technique used was adequate to identify nematode isolates, enabling knowledge of its biodiversity and contributing to the detection of new isolates that may be used in biological control programs of insect pests.

The sequence of D2/D3 expansion 28S rDNA gene analysis by DNA barcode technique was useful for the diagnosis of H. *amazonensis, S. rarum, M. rainai* and *O. tipulae*. The phylogenetic tree obtained from the 48 aligned sequences of the expansion D2/D3 EPNs with four distinct groups support the molecular identification of these nematodes isolated from soil samples. This technique has been used to diagnose plant and animal parasites and entomopathogenic nematodes with accurate and reliable results, such as one *Pratylenchus penetrans* (Cobb) specimen in potato^41^, *Bursaphelenchus fungivorus* (Franklin & Hooper) in coconut fiber^43^, *M. rainai* (Carta & Osbrink) in soil cultivated with soybean^44^ and *Metarhabditis blumi* (Sudhaus) parasitizing the ear canal of cattle^45^.

The finding of *H. amazonensis* and *S. rarum* in seven (3.5%) of the 201 soil samples, shows the reduced occurrence of these organisms in Brazil compared to surveys in Western Canada (20%)^46^, Argentina (13.2%)^47^ and Spain (23.3%)^48^. However, the prevalence of 3.5%, of these species, is similar to surveys in Turkey (2%, 9.1%)^25^,^49^, Azores Archipelago, Portugal (3.9%)^19^ and Minas Gerais state, Brazil (9%)^29^. The absence of EPNs in plowed soil areas suggests inadequate conditions for nematode survival, but zero EPN detection in the natural forest was unexpected and may be due the low soil samples collected in this area. This result may also indicate the need for a higher number of samples taken at different soil depths. The species habitat and soil type affected EPNs recovery^25^,^50^. Our samples with nematodes were obtained from, clay (75%) and sandy (25%) soils, indicating the mobility and survival of EPNs in soils rich in sand, but *S. rarum* occurred in clay (FCA 09 and FCA 10) and sandy (FCA, 11, 12, 13 and 14) soils. Many EPNs positive samples (89.65%) were obtained in acid soils in Nepal^51^. Six EPNs were found in soils with PH < 4, which is uncommon, but this has also been reported in Belgium^52^. *Steinernema* spp. are widespread in different regions and niches, whereas *Heterorhabditis* spp. are common in forest and river banks^53^. *Heterorhabditis* and *Steinernema* species were found in Sandy soils with pH < 6, whereas representatives of *Heterorhabditis*, mainly in Sandy soils with pH > 6 were found on nine islands of Açores^19^.

*Steinernema rarum* detection in sandy soil with *Rubus edaeus, Mangifera indica* and *Oryza sativa* crops and clay soils with *Citrus* sp. and *Eucalyptus* sp. demonstrates the habitat diversity of this nematode, which has been detected in *Olea europea* L. crops in Argentina^54^, *Carya illinoinensis* orchards (Wangenh.) in Mississippi and Louisiana State, USA^28^ and in sandy soils with annual and forest cultures in China^55^. In Brazil, this nematode was detected in soils with *Nicotiana tabacum* L., *Triticum aestivum* L., *Glycine max* and in native vegetation^56^. *Steinernema rarum* showed desiccation tolerance and freezing intolerance^57^, features that may be related to its adaptation to Brazilian soils. This species has shown promise in biological control programs against *Antonomus grandis* Boheman (Coleoptera: Curculionidae) and *Spodoptera frugiperda* J.E. Smith (Lepidoptera: Noctuidae)^27^. *Metarhabditis rainai* detection in fruit orchards, annual crops and forest areas shows the broad habitat diversity of this nematode. This species was reported in *Glycine max* crop in Araras, São Paulo State, Brazil^44^. Other species, such as *Metarhabditis blumi*, are considered entomopathogenic and associated with the bacteria *Alcaligenes faecalis* Castellani & Chalmers, *Flavobacterium* sp. (Bernardet & Grimont) and *Providencia vermicola* (Somvanshi), which killed *G. mellonella* larvae^58^. Other genera, besides *Heterorhabditis* and S*teinernema* may feed on and kill insects^44^. Rhabditidae nematodes are usually bacteriophages, found in insect carrions^59^. However, *M. rainai* was described from specimens isolated from the *Oscheios tipulae* termite gut^60^ detected in soil samples and may parasitize insects and was also found to be associated with *Tipula paludosa* Meigen (Diptera: Tipulidae) larvae^61^. This nematode has been frequently isolated in soil samples from around the world, enabling species population^62^. *Oscheius* genus (Rhabditidae) includes free-living, vertebrate and invertebrate parasitic species^63^, such as *Oscheios chongmingensis* (Zhang), considered a facultative entomopathogenic nematode^64^. The bacteria symbiosis with this genus species is similar to that of Steinernematidae and Heterorhabditidae^65^.

EPNs identified in agricultural areas in Brazil were *Heterorhabditis amazonensis, Metarhabditis rainai, Oscheios tipulae* and *Steinernema rarum*. These species should be considered a pest biocontrol agent.

## Additional Information

**How to cite this article**: de Brida, A. L. *et al*. Entomopathogenic nematodes in agricultural areas in Brazil. *Sci. Rep.* **7**, 45254; doi: 10.1038/srep45254 (2017).

**Publisher's note:** Springer Nature remains neutral with regard to jurisdictional claims in published maps and institutional affiliations.

## Figures and Tables

**Table 1:** Code (Code), species, sampling site (Site), soil type (S), crops, geographical coordinates (coordinates) and collection date (date) of entomopathogenic nematodes (species) in soils with annual, fruit and forest plantation crops in Brazil (2010 to 2013).

Code	Species	Site	S	Crops	Coordinates	Date
FCA01	*M. rainai*	Bo (SP)	C	*Citrus*	S773173; W7484343	Oct/2011
FCA02	*O. tipulae*	Bo (SP)	C	*Citrus*	S773250; W7484319	Oct/2011
FCA03	*O. tipulae*	Pa (PR)	C	*G. max*	S4172381; W3521906	Jan/2013
FCA04	*M. rainai*	Ga (SP)	C	*A. fraxinifolius*	S763062; W77469943	Jan/2013
FCA05	*O. tipulae*	Ga (SP)	C	*A. indica*	S640610; W7544268	Jan/2013
FCA06	*M. rainai*	Ga (SP)	C	*C. odorata*	S640581; W7544259	Jan/2013
FCA07	*H. amazonensis*	Ga (SP)	C	*P. parviflora*	S640551; W7544214	Jan/2013
FCA08	*M. rainai*	Ga (SP)	C	*C. brasiliense*	S640467; W7544384	Jan/2013
FCA09	*S. rarum*	Bo (SP)	C	*Citrus*	S766468; W7474631	Sept/2012
FCA10	*S. rarum*	Bo (SP)	C	*Eucalyptus*	S764574; W7472130	Sept/2012
FCA11	*S. rarum*	Bo (SP)	S	*O. sativa*	S766897; W7474367	Sept/2012
FCA12	*S. rarum*	SM (SP)	S	*R. idaeus*	S749498; W7479197	Oct/2012
FCA13	*S. rarum*	SM (SP)	S	*Citrus*	S749477; W7479125	Oct/2012
FCA14	*S. rarum*	SM (SP)	S	*M. indica*	S749436; W7479022	Oct/2012
FCA15	*M. rainai*	Ga (SP)	C	*C. ecalyculata*	S640534; W7544537	Jan/2013
FCA16	*M. rainai*	Pa (PR)	C	*G. max*	S4211166; W3491260	Jan/2013

Species: *Metarhabditis (M.*), *Oscheius (O.*), *Heterorhabditis (H.*), *Steinernema (S.*), Site: Botucatu (Bo), Palotina (Pa), Garça (Ga), São Manuel (SM), clay soil (C), Sandy soil (S). Date: Octuber (Oct), January (Jan), September (Sept).
